# Chronic hyperventilation with hypocapnia: the unstable face of ventilatory control

**DOI:** 10.36416/1806-3756/e20260319

**Published:** 2026-11-09

**Authors:** Mateus Dutra Batalha Costa, Mariana Lima Lafetá, Eloara V M Ferreira

**Affiliations:** 1. Divisão de Pneumologia, Escola Paulista de Medicina, Universidade Federal de São Paulo, São Paulo (SP) Brasil.

## INTRODUCTION

Many individuals who experience unexplained breathlessness present with irregularities in breathing patterns and control.^1^ Dysfunctional breathing is defined as “a respiratory disorder, psychologically or physiologically based, involving breathing too deeply or too rapidly”.^(REF)^ Hyperventilation syndrome (HVS) is the most common form, with a prevalence estimated at 6-10% in the general population.^2^ It is often associated with shortness of breath, chest tightness, lightheadedness, and paresthesia.^(REF)^ Cardiopulmonary exercise testing (CPET), especially with blood gas analysis (CPET level 2), can be an attractive method for identifying different types of dysfunctional breathing patterns.

## OVERVIEW

A 44-year-old, healthy, professional woman presented with persistent dyspnea (modified Medical Research Council dyspnea scale score of 3) after a mild case of COVID-19. Her medical history included asthma, obesity, and anxiety. The results of initial evaluations, including spirometry, echocardiography, Holter, and chest CT angiography, were unremarkable. To investigate her dyspnea, CPET with ear-lobe arterialized blood gases was performed (Figure 1A). The results demonstrated preserved aerobic capacity and a normal cardiocirculatory response. However, there was increased ventilatory drive, indicated by an elevated ventilatory equivalent for CO_2_ (V̇_E_/V̇CO_2_) and a markedly reduced end-tidal expiratory CO_2_ pressure throughout the CPET. Blood gas analysis confirmed respiratory alkalosis (high pH and low PaCO_2_), preserved dead space to tidal volume (V_D_/V_T_) ratio, and a normal alveolar-arterial O_2_ gradient. An erratic breathing pattern was observed, at rest and during exercise. The following equation gives the minute ventilation (V̇_E_) adjustment: 

**Figure 1:**
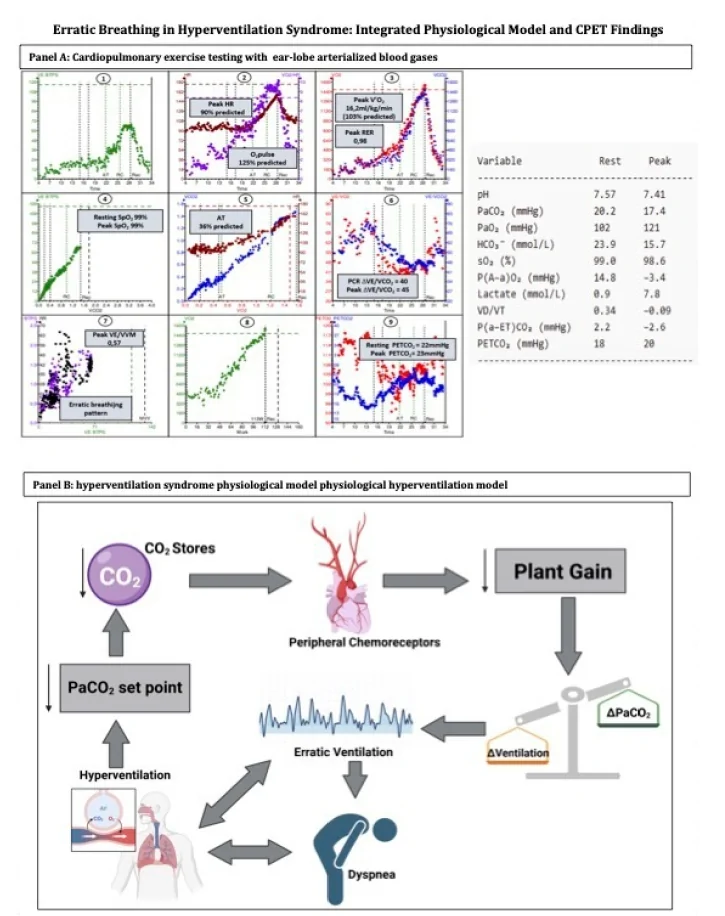
Integrated assessment of erratic breathing in hyperventilation syndrome. Panel A: Cardiopulmonary exercise testing (CPET, level 2) demonstrates the following features consistent with hypocapnia and respiratory alkalosis: subpanel 3-preserved aerobic capacity; subpanel 7-erratic breathing pattern with increased ventilatory drive; subpanel 6-elevated ventilatory equivalents; and subpanel 9-reduced end-tidal expiratory CO_2_ pressure (PETCO_2_). Arterialized blood gas analysis confirms low PaCO_2_ at rest and during exercise with preserved oxygenation and normal alveolar-arterial gradient. Panel B: The pathophysiological model illustrates how chronic hyperventilation reduces CO_2_ stores and lowers the PaCO_2_ set point, impairing buffering capacity and increasing ventilatory instability. Enhanced peripheral chemoreceptor sensitivity and reduced plant gain (ΔPaCO_2_/ΔV̇_E_ ratio) amplify the ventilatory response to small fluctuations in PaCO_2_, leading to erratic breathing despite preservation of the cardiopulmonary structure.

$$\dot{V}E=\;863\;\times VCO2∕\;\left(PaCO2\times \;\left[1\;-VD/VT\right]\right)$$

where V̇_E_ is proportional to the muscle metabolism (V̇CO_2_) and the V_D_/V_T_ ratio, as well as being inversely correlated with the PaCO_2_. It is well known that CO_2_ plays a critical role in physiological buffering, and, at sea level, PaCO_2_ is typically maintained at approximately 40 mmHg and remains unchanged during moderate exercise.^3^ ^,^ ^4^ As part of the spectrum of dysfunctional breathing disorders, HVS is associated with increased ventilatory drive and compensated respiratory alkalosis, with a normal or slightly elevated pH and a low PaCO_2_ (typically < 30 mmHg). Patients with dysfunctional breathing tend to present with a high respiratory rate at rest, which inappropriately increases during exercise, while V_T_ may remain relatively unchanged or erratic. Persistent hyperventilation at rest changes the magnitude of the ventilatory response, requiring a new PaCO_2_ set point at a lower CO_2_ level, perpetuating a vicious circle of metabolic acid-base imbalance and heightened peripheral chemoreceptor sensitivity (altered CO_2_ chemosensitivity, CO_2_ store depletion, and reduced plant gain, which is defined by the ΔPaCO_2_/ΔV̇_E_ ratio and reflects the extent to which the PaCO_2_ changes in response to variations in ventilation. When it is altered, a given change in PaCO_2_ can result in markedly different effects on ventilation,^5^ as illustrated in Figure 1B. In HVS, the peripheral sensitivity range shows increased tonic activity at rest, resulting in an elevated ventilatory drive. Over time, this pattern can result in chronic hyperventilation, reducing total-body CO_2_ stores and diminishing the ability of the body to stabilize ventilatory fluctuations. Even minor variations in PaCO_2_ can lead to larger, more rapid changes in ventilation, increasing system instability. These alterations interact in a self-reinforcing manner, sustaining the cycle of chronic hyperventilation.^4^ ^,^ ^5^ These findings provide a mechanistic explanation for the clinical features of HVS, including an erratic breathing pattern, symptom exacerbation in response to minor stimuli, and disproportionate dyspnea. In addition, patients with HVS are not expected to have exercise-induced hypoxemia or ventilatory inefficiency, given that the underlying issue is related to ventilatory drive. This aspect helps differentiate HVS from other causes of chronic hyperventilation, such as heart failure and pulmonary arterial hypertension. 

## CLINICAL MESSAGE

For evaluating breathing patterns and gas exchange, CPET with blood gas analysis is a valuable tool. An erratic breathing pattern accompanied by hyperventilation, with or without hypocapnia, strongly suggests dysfunctional breathing. While this may be triggered by stress or anxiety, it can also result from an underlying cardiopulmonary disease.
